# A polar bundle of flagella can drive bacterial swimming by pushing, pulling, or coiling around the cell body

**DOI:** 10.1038/s41598-017-16428-9

**Published:** 2017-12-01

**Authors:** Marius Hintsche, Veronika Waljor, Robert Großmann, Marco J. Kühn, Kai M. Thormann, Fernando Peruani, Carsten Beta

**Affiliations:** 1 University of Potsdam, Institute of Physics and Astronomy, 14476 Potsdam, Germany; 2 Université Côte d’Azur, Laboratoire J. A. Dieudonné, UMR 7351 CNRS, F-06108 Nice Cedex 02, France; 3 Institut für Mikrobiologie und Molekularbiologie, Justus-Liebig-Universität Giessen, 35392 Giessen, Germany

**Keywords:** Cellular motility, Bacteria, Biological physics

## Abstract

Bacteria swim in sequences of straight runs that are interrupted by turning events. They drive their swimming locomotion with the help of rotating helical flagella. Depending on the number of flagella and their arrangement across the cell body, different run-and-turn patterns can be observed. Here, we present fluorescence microscopy recordings showing that cells of the soil bacterium *Pseudomonas putida* that are decorated with a polar tuft of helical flagella, can alternate between two distinct swimming patterns. On the one hand, they can undergo a classical push-pull-push cycle that is well known from monopolarly flagellated bacteria but has not been reported for species with a polar bundle of multiple flagella. Alternatively, upon leaving the pulling mode, they can enter a third slow swimming phase, where they propel themselves with their helical bundle wrapped around the cell body. A theoretical estimate based on a random-walk model shows that the spreading of a population of swimmers is strongly enhanced when cycling through a sequence of pushing, pulling, and wrapped flagellar configurations as compared to the simple push-pull-push pattern.

## Introduction

Swimming motility is ubiquitous in the living world. It is essential for many biological functions, such as the search for nutrients, sexual reproduction, or the spreading of diseases^1^. Among microbial swimmers, bacteria are one of the largest and most prominent classes^2^. They propel themselves with the help of helical flagella that are connected to rotary motors in their cell wall. Bacteria typically move in a sequence of persistent runs that are interrupted by random reorientation events. The most thoroughly studied example, the intestinal bacterium *Escherichia coli*, displays several flagella distributed across its cell envelope (peritrichous flagellation). Upon counterclockwise rotation of their motors, they form a coherent flagellar bundle that pushes the cell forward in a run. When one or several of the motors reverse their sense of rotation, the bundle disassembles and a random reorientation of the cell body occurs (tumble), setting the direction of the next run^2,3^.

Over the past years, it became clear that, depending on the number and the arrangement of flagella, a variety of swimming patterns may emerge that differ from the classical run-and-tumble strategy of *E. coli*. For example, bacteria that are decorated with a single flagellum at their cell pole (monotrichous flagellation) typically display sharp reversals in their swimming direction^4,5^, but may also exhibit more complex maneuvers, such as the run-reverse-flick pattern of *Vibrio sp*.^6,7^. We have recently concentrated our research on the soil bacterium *Pseudomonas putida* ^8^ that propels itself with a tuft of helical flagella attached to the posterior pole of its elongated cell body (lophotrichous flagellation)^9^. Similar to monotrichous bacteria, *P. putida* swims in straight runs that are interrupted by sharp reversals in the swimming direction. But in addition, also events with small turning angles centered around *θ* = 0° occurred. In contrast to other bacterial swimmers, *P. putida* may also change its swimming speed between two different levels when reversing its swimming direction. However, not every reversal is associated with a speed change.

These findings pose several fundamental questions regarding the swimming strategy of *P. putida*. Monotrichous bacteria induce reversal events by changing the sense of rotation of their flagellar motor. These reversals are associated with a transition from pushing to pulling. However, in the case of bacteria with multiple flagella, the pulling mode may be obstructed by instabilities and jamming of the pulling flagellar bundle^10,11^. Does *P. putida* exhibit a stable pulling bundle or is the reversal event related to a different type of flagellar arrangement? For example, we envisioned that *P. putida* flips the orientation of its flagellar filaments, so that they point backward even though the flagellated pole of the cell is oriented in swimming direction^12^. Also the origin of the two different swimming speeds remains unclear. To address these questions, we performed high speed fluorescence recordings of the flagellar dynamics of swimming *P. putida* cells.

## Results

Our fluorescence imaging experiments revealed that *P. putida* can propel itself in three distinct swimming modes: the flagellar bundle can push or pull the cell, or it may wrap around the cell body to initiate propagation of the cell in a screw-like fashion. Animations displaying examples of all three swimming modes are provided as Supplementary Videos, see the detailed explanations below.

### Swimming as a pusher

**Figure 1:**
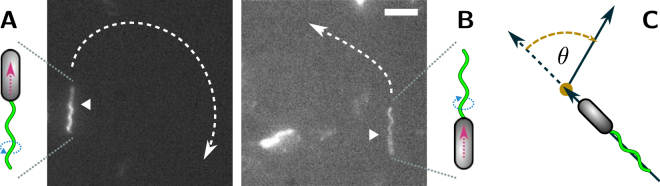
*P. putida* can swim (**A**) as a pusher and (**B**) as a puller. In the vicinity of a solid interface, trajectories of pushers turn to the right, whereas trajectories of pullers turn left. The posterior (flagellated) cell pole is marked by a white triangle. Scale bar is 5 *μ*m. See also the corresponding Supplementary Videos S1 and S2. (**C**) Definition of the turning angle *θ*.

In the majority of cases, *P. putida* was observed to move as a pusher, with the rotating flagellar bundle driving the motion from the rear end of the cell body, see Fig. 1A and Supplementary Video S1. Typically, the flagella appeared closely assembled into a coherent bundle. But we also observed cases, where cells were driven by a loose, open tuft of individual filaments, see Supplementary Video S7. The trajectories of pushers were either straight or curved to different degrees. In the case of curved trajectories, pushers always turned to the right.

As our data is recorded in close proximity to the microscope cover slip, curvature in the swimming trajectories can thus be attributed to a well-known hydrodynamic wall effect: In the vicinity of solid surfaces, bacterial swimmers experience a wall-induced torque that results in a bending of the otherwise straight swimming path, see^13^ and references therein. From our observation that pushing *P. putida* cells invariably turn to the right, we can conclude that pushers are driven by a left-handed helix turning in counterclockwise direction, see^14^ for details.

### Swimming with a stable pulling bundle

To a much lesser extent, we also observed *P. putida* cells that moved as pullers. Here, the flagellated posterior pole of the cell body was oriented in swimming direction with the rotating bundle pointing ahead, see Fig. 1B and Supplementary Video S2. From our fluorescence recordings, it is often difficult to decide, whether a cell is propelled by a single flagellum or by a tightly assembled, coherent bundle. However, in cases where motion is interrupted by pauses in the motor rotation, bundles typically disassemble, revealing that they are composed of several individual flagella, for an example see Supplementary Video S3. Based on these observations, we unequivocally confirmed that *P. putida* can propel itself with a stably pulling flagellar bundle.

As was the case with pushers, we also observed a bias in the curvature of the trajectories for pullers. They were either straight or displayed a more or less pronounced bending to the left, indicating that pullers swim by turning a left-handed helical bundle in clockwise direction^13^. The rare occurrence of pullers in our recordings can be also attributed to hydrodynamic interactions between the swimmer and the adjacent cover slip. While pushers are hydrodynamically stabilized in parallel orientation to the surface, pullers are preferentially driven into a direction perpendicular to the wall^13^. They are thus less likely to be detected in our recordings.

### Swimming with the flagella wrapped around the cell body

**Figure 2:**
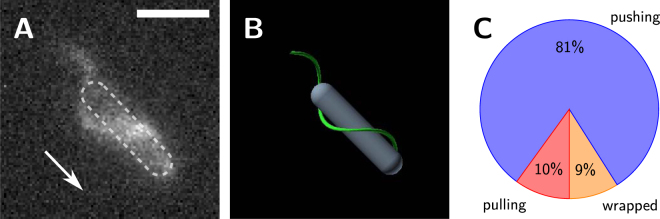
*P. putida* can swim with the flagellar bundle wrapped around the cell body. (**A**) Fluorescence image and (**B**) cartoon of the wrapped mode. The cell swims in the direction indicated by the arrow – the posterior (flagellated) pole of the cell points in swimming direction, see also the Supplementary Videos S4 and S5. (**C**) Percentages of swimming states observed in our data set (out of 794 runs in total). Scale bar is 5 *μ*m.

Across our entire data set, we observed numerous examples of cells that swam with their filament bundle wrapped around the cell body, resembling a drill or corkscrew geometry, see Fig. 2 and Supplementary Video S4. Cells moved with the posterior pole pointing in the direction of motion and circled their helical bundle around the cell body to screw their way forward though the viscous fluid environment. Note that a similar flagellar arrangement has been recently reported for monotrichous cells of *Shewanella putrefaciens* ^15^.

When wrapped around the cell body, the flagellar bundle took the form of a left-handed helix that turned in clockwise direction, as could be directly observed in our recordings, see Supplementary Video S5. Even though recorded in the vicinity of a cover slip, we could not detect any systematic bias in the curvature of the trajectories of cells that swam in the wrapped mode as these trajectories were mostly straight. It is well known that bacterial flagella can switch between different polymorphic states as a result of their internal structure^16,17^. The polymorphic states differ in their geometric parameters such as handedness, radius, and pitch of the helix^18,19^. As the diameter of the helical bundle in the wrapped mode was much larger than in pushing or pulling mode, we assume that also in this case, the flagella adopt different polymorphic states: a stretched left-handed conformation with small diameter during pushing and pulling that we call the normal state (radius: 0.4 ± 0.1 *μ*m, pitch: 2.0 ± 0.1 *μ*m), and a more compact left-handed conformation with larger diameter when wrapped around the cell body that we refer to as the coiled state (radius: 0.6 ± 0.1 *μ*m, pitch: 2.1 ± 0.3 *μ*m).

### When wrapped around the cell body, swimming speed and motor frequency differ from the pushing and pulling modes

We have analyzed the swimming speed of cells that moved with their flagella wrapped around the cell body and compared it to the speeds measured for pushers and pullers. While we could not detect any speed differences between pushers and pullers, the cells that moved in the wrapped mode were significantly slower. In particular, we measured 15.5 ± 0.2 *μ*m/s in wrapped mode, 25.4 ± 0.4 *μ*m/s in pushing, and 29.0 ± 1.5 *μ*m/s in pulling mode. The Wilcoxon rank sum test indicated that the median of pushing and pulling run speeds was significantly different (p = 0.05) compared to the speed of runs in wrapped mode, see also Supplementary Fig. S1.

The different speeds are related to differences in the rotation frequency of the motors between the push/pull states and the wrapped state. In freely swimming cells, the motor frequency is equal to the difference between the frequencies of filament rotation and cell body rotation, *ω*_m_ = *ω*_f_ − *ω*_b_, where *ω*_f_ and *ω*_b_ have opposite sign. As we cannot directly extract the body rotation frequency *ω*_b_ from our data, the motor frequency *ω*_m_ is difficult to access from freely swimming cells. However, in our data sets, we encountered several examples of cells that got stuck to the cover slip but still maintained a fully operational rotary bundle performing sequences of transitions between the different bundle configurations. In these cases, the cell body did not rotate (*ω*_b_ = 0), so that the rotation frequency of the motors equals the rotation frequency of the filaments, *ω*_m_ = *ω*_f_, which could be directly measured. From the recordings of surface attached cells, we estimated a motor rotation frequency of 200 Hz in the push/pull mode and 50 Hz in the wrapped mode.

We furthermore note that movement in all three swimming modes was from time to time interrupted by stops in the motor rotation. Some of these pauses were very short, barely affecting the course of locomotion. But rotation may also cease for longer episodes resulting in a halt of the cell body and a gradual disassembly of the flagellar bundle, see for example Supplementary Videos S6 and S7. We will now turn to the transition scenarios between the different swimming modes, where the flagellar bundle is pushing, pulling, or wrapped around the cell body.

### Transition between pushing and pulling mode

Cells can reverse their swimming direction by a sudden switch from pushing to pulling and vice versa, see for example Supplementary Videos S3 and S8. Here, all motors synchronously switch their direction of rotation. Even though the temporal resolution of our imaging data is not sufficient to directly visualize the sense of motor rotation in pushing and pulling mode, the handedness of the flagella and the direction of motor rotation are uniquely determined by the curvature of trajectories in the vicinity of a solid boundary as described above. We can thus safely conclude that push-pull transitions are associated with a switch in the sense of rotation of the left-handed helical bundle.

### Transition from a pulling to a wrapped bundle

**Figure 3:**
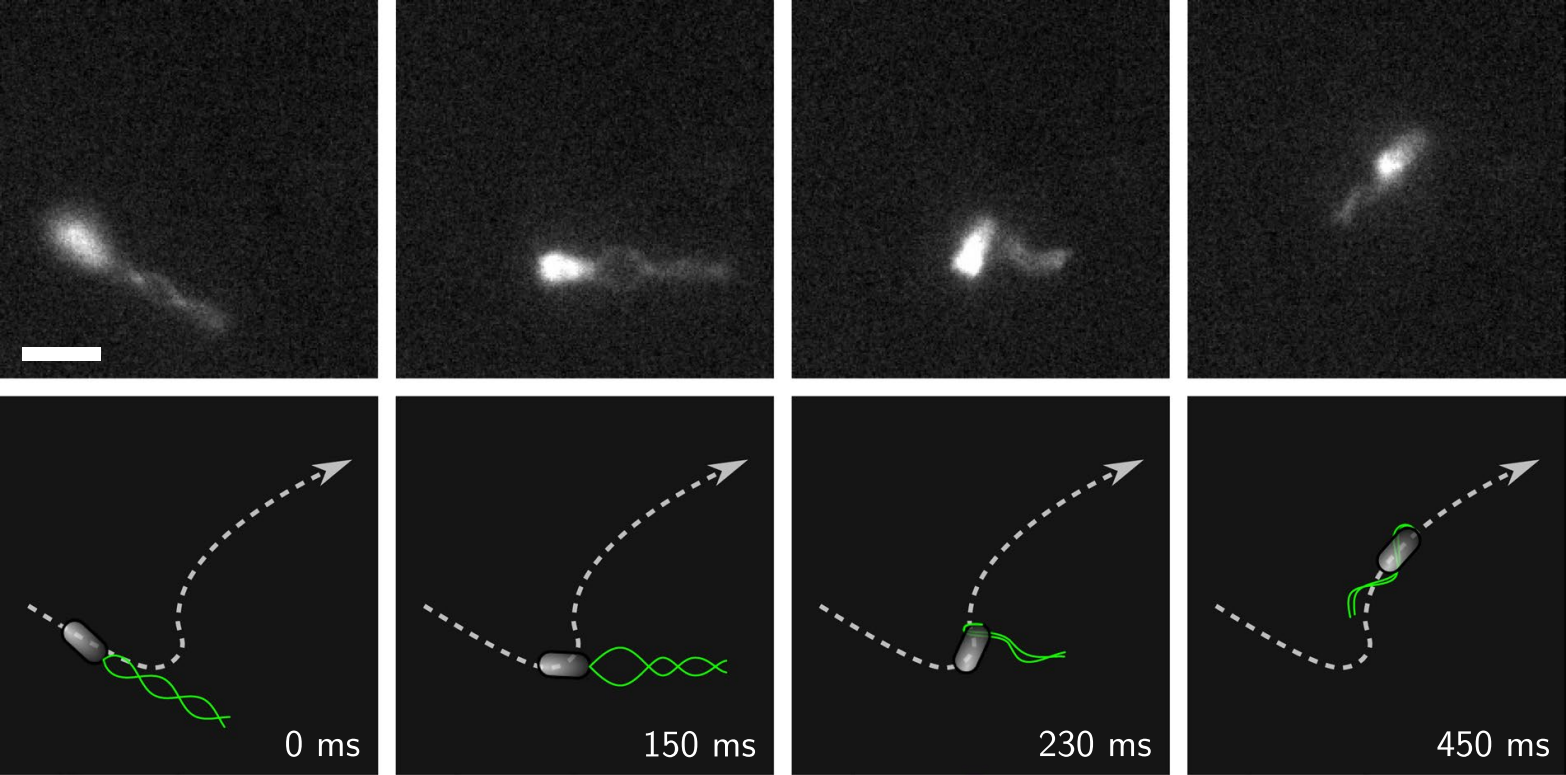
Transition from a pulling to a wrapped bundle. (Top) Snapshots of the transition from a fluorescence microscopy recording, see also the corresponding Supplementary Video S9. Scale bar is 3 *μ*m. (Bottom) Cartoon of the transition and cell trajectory (dashed line).

When swimming as a puller, *P. putida* can wrap its flagellar bundle around the cell body. The transition is initiated by a strong bending of the bundle close to the point where it is attached to the cell pole. The bundle then coils around the cell body and the cell moves on in a corkscrew fashion as described above. A sequence of intermediate flagellar configurations that occur in the course of this transition is displayed in Fig. 3, see also the corresponding Supplementary Video S9. In both swimming modes, the flagellated pole of the cell body points in swimming direction. Consequently, the turning angle *θ* of the transition from a pulling to a wrapped bundle was observed to be small and centered around zero.

More detailed recordings of this transition could be obtained for cells that accidentally stuck to the microscope cover slip. In this case, the flagellar dynamics could be observed over a longer period of time including several transitions between the different swimming states, see Supplementary Fig. S2 and Supplementary Video S10. Note that the back transition from a wrapped to a pulling bundle was not observed in our recordings.

### Transition from a wrapped to a pushing bundle

**Figure 4:**
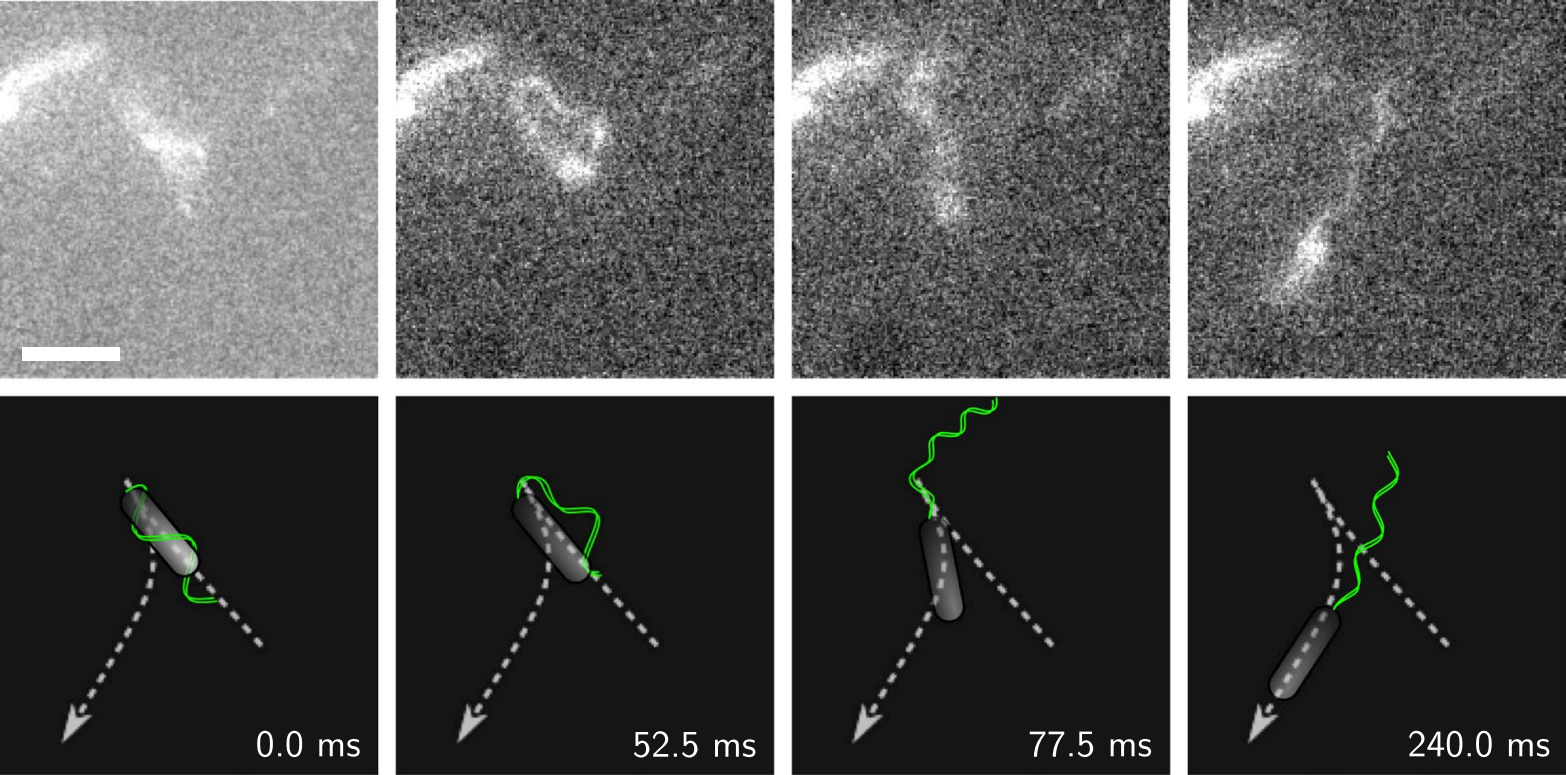
Transition from a wrapped to a pushing bundle. (Top) Snapshots of the transition from a fluorescence microscopy recording, see also the corresponding Supplementary Videos S11. Note that in the upper left corner a second, non-motile bacterium can be seen. Scale bar is 3 *μ*m. (Bottom) Cartoon of the transition and cell trajectory (dashed line).

When swimming with a wrapped bundle, *P. putida* can unfold its flagella from the close coiling around the cell body to undergo a transition into the pushing mode. Again, the transition originates from the proximal end of the bundle. This time, it is initiated by a change in the direction of bundle rotation. The bundle is pushed in counterclockwise direction, so that close to the hook a loop opens and grows between the cell body and the bundle. Finally, the entire bundle stretches out in a rapid whip-like stroke, and the cell moves on as a pusher. An example can be seen in the sequence of images displayed in Fig. 4, see also the corresponding Supplementary Videos S11. Also for this transition, we additionally provide a selection of intermediate bundle configurations from an immobilized cell in Supplementary Fig. S3 and Supplementary Video S12.

Before the transition, in the wrapped mode, the flagellated pole of the cell body pointed in swimming direction. After unfolding of the bundle, when the cell moves as a pusher, the flagellated pole is oriented away from the swimming direction, i. e., the cell has reversed its direction of motion. Consequently, the turning angle of this transition is large and centered around *θ* = 180°. Note that the back transition from a pushing to a wrapped bundle was not observed in our recordings.

## Discussion

Based on fluorescent staining of the flagella in combination with high-speed imaging, we could show that the soil bacterium *Pseudomonas putida*, which drives its swimming motion with a polar tuft of helical flagella (lophotrichous flagellation), can move as a pusher, as a puller, or with its bundle wrapped around the cell body.

### A stable lophotrichous puller

While propagation as a pusher is well-known from many different bacteria, clear evidence for the pulling mode based on high-resolution fluorescent imaging of the flagella has so far only been reported for monotrichously flagellated species. Older reports on the swimming behavior of lophotrichously flagellated *Chromatium okenii* are in favor of a pulling filament configuration^20^ but the stability of pulling flagellar bundles has been questioned by theoretical arguments and experiments on macroscopic helical bundles^10,11^. In contrast, our results show that the helical bundle of a lophotrichously flagellated *P. putida* cell may also function in a stable pulling configuration.

In peritrichously flagellated bacteria, motors are only weakly coupled by diffusion and normally only few flagella participate in a tumble^21^. Here however, all motors appear to switch their sense of rotation synchronously during a push-pull transition and also transitions from and into the wrapped state apparently happen at the same time. Therefore, it will be of interest to look for the mechanism which enables this tight coupling of flagellar motors. Their close physical proximity might indeed play a role, since other protein clusters are known to exhibit coordinated responses to input cues in order to integrate them^22–25^.

### Free swimming with a wrapped bundle in bulk fluid

While pushing and pulling configurations have been observed for various other bacterial swimmers, the wrapped mode, where the helical bundle is tightly coiled around the cell body, has only recently been reported for monotrichous *Shewanella putrefaciens* ^15^ and was not observed for lophotrichous bacteria before. However, also other bacterial species may use this type of motility as recent observations on *Magnetospirillum magneticum* indicate^26^. During this novel type of swimming propulsion, the cell moves like a corkscrew through the viscous environment. For reasons of torque balance, the helical bundle and the cell body inside the bundle have to rotate in opposite directions. This will result in a surrounding flow field that differs from the classical dipole fields of pushers and pullers, see^13^ and references therein. In agreement with this, we observed that the hydrodynamic wall interactions of cells with wrapped flagella differ from those of conventional pushers and pullers. While pushers and pullers experience a torque when moving close to a solid boundary, the trajectories of cells with wrapped flagella were observed to be straight. More detailed experimental and theoretical studies are required to elucidate the flow field of this novel type of swimmer.

For *S. putrefaciens* it was demonstrated that a wrapped filament configuration promotes the escape of cells that are trapped in narrow spacings^15^. In particular, it facilitates the release from confinement by enabling a screw-like backward motion of cells. Consequently, it was observed under conditions of confinement and in conjunction with surface interactions^15^. In contrast, *P. putida* regularly switches into the wrapped flagellar configuration also during free swimming in bulk fluid. This can be concluded from a comparison of swimming speeds. Our fluorescence recordings show that in wrapped mode, the swimming speed is reduced by a factor of two on average. This correlates well with earlier cell tracking experiments in bulk fluid, where similar changes in the swimming speed were observed^8^.

The experiments with monotrichous *S. putrefaciens* have furthermore shown that in the bulk fluid, a transition to the wrapped mode is more likely to occur for an increased viscosity of the surrounding liquid^15^. We conjecture that in the case of a lophotrichous swimmer, the close proximity and the resulting mechanical interactions between several polar filaments can be regarded, in first approximation, as an increased effective viscosity that each filament will experience. We may thus speculate that in the bulk fluid, a transition to the wrapped mode occurs more commonly for lophotrichous than for monotrichous bacteria as a consequence of the different flagellation pattern. Note that also for *Pseudomonas fluorescens* phases of slow swimming (backup) have been reported^27^, suggesting that also in this case, the flagellum may assume a wrapped configuration.

### A change in motor torque initiates wrapping of the flagellar bundle

**Figure 5:**
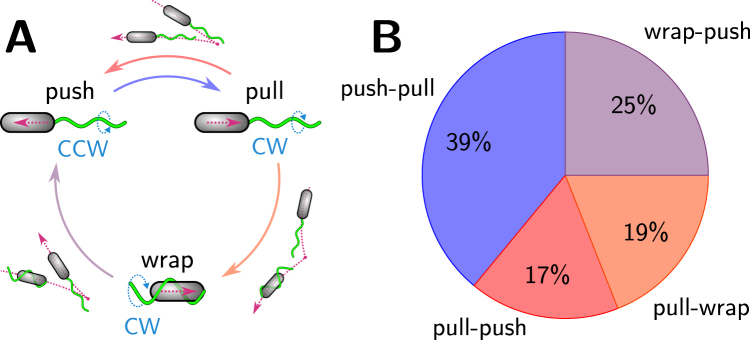
Swimming pattern of *P. putida*. (**A**) Cycle of transitions with the two alternative swimming patterns, push-pull and push-pull-wrapped. Transitions between the swimming states are indicated by colored arrows (colors correspond to the color coding used in (**B**)). A cartoon next to the arrows depicts the resulting trajectories. (**B**) Percentages of transitions observed in our data set (out of 80 transitions in total).

The transitions that we observed between the three swimming modes are summarized in Fig. 5. In all three states, the flagellar bundle takes the form of a left-handed helix that can turn in counterclockwise (pushing) or clockwise direction (pulling and wrapped). The push-pull transition was found to be reversible. For the transitions from a pulling to a wrapped bundle and from a wrapped to a pushing bundle the corresponding back transitions were not observed in our data.

We note that in both the pulling and the wrapped configuration, the flagellar bundle is rotating in clockwise direction. Thus, the transition from a pulling to a wrapped bundle is not associated with a switch in the sense of rotation of the filament bundle but is initiated solely by a change in the motor torque. This is remarkable as all of the known bacterial turning scenarios, such as tumbles, reversals, or flicks, involve reversals in the direction of motor rotation, or at least a complete stop. Also the polymorphic state of the flagella is different in both modes. In the pulling and pushing modes, we observed a stretched left-handed helix with small diameter, whereas in the wrapped mode the bundle assumed a more compact left-handed helical form with larger diameter. Due to the qualitative similarities to known polymorphic forms of other bacterial flagella, we refer to them as the normal and coiled states, respectively^28^. Theoretical modeling showed that the polymorphic state may change depending on the motor torque^29^. However, in the case of *P. putida*, the transition from a pulling to a wrapped bundle may also occur in cases, where it is not accompanied by a change in the polymorphic state and the filament bundle displays the coiled configuration before and after the transition, see Supplementary Video S13.

For monotrichously flagellated bacteria the transition to the wrapped bundle was explained on the basis of an instability that may occur during pulling. If stress on the pulling filament increases due to enlarged friction on the cell body or increased motor torque, an incomplete perversion of the helical filament close to the cell body may trigger wrapping of the filament around the cell body^15^. A similar scenario may also hold for the helical bundle of a lophotrichous swimmer. In contrast, the transition from a wrapped to a pushing bundle is associated with a change in the sense of motor rotation, similar to the transitions between the pushing and pulling mode and in line with other known bacterial turning scenarios. This suggests that for the wrapped flagellar geometry, cells that move with their flagellated pole pointing in swimming direction are the only stable configuration.

### A swimming strategy with two alternative routes

From the transitions displayed in Fig. 5 it becomes clear that *P. putida* may alternate between two different swimming strategies. It can either move in a classical push-pull-push pattern, similar to many monotrichously flagellated marine bacteria^5^, or it may propel itself in a cycle passing through pushing, pulling, and wrapped bundle configurations. Also mixtures of both patterns are possible. In this picture, the critical decision between one or the other route is taking place after a pulling phase, when the cell may either turn back to the pushing mode or switch into the wrapped mode.

How is the overall spreading of a population of swimmers affected if an additional phase of slow swimming with wrapped flagella occurs between the pulling and pushing phase? The diffusion coefficients for several run-and-turn scenarios have been previously calculated^30,31^. Here, based on a model for actively moving particles in three dimensions^32^, we estimated the diffusion coefficient of swimmers that cycle through pushing, pulling, and wrapped modes. Our estimate is based on the following simplifying assumptions. We assume that run times are exponentially distributed with a mean run time *τ*, so that transitions between the swimming states occur at a rate of *k* = 1/*τ*. Based on our earlier tracking results^8^, we take *k* to be the same for all three swimming modes and neglect the duration of turning maneuvers. The transitions between pushing and pulling and between wrapped and pushing mode are accompanied by reversal events (average turning angle of *θ* = 180°), whereas the transition from pulling to wrapped mode does not involve a reversal event (average turning angle of *θ* = 0°). The speed in the wrapped state is assumed to be a factor of two smaller compared to the push and pull states. We also include rotational diffusion during the runs, which is due to thermal fluctuations and intrinsic noise in the cellular propulsion machinery. In addition, we introduce the splitting probability *p* that connects the two alternative swimming patterns. When leaving the pulling mode, the cell will enter the wrapped mode with a probability *p* or switch back to the pushing mode with probability 1 − *p*.

To derive the diffusion coefficient depending on the splitting probability *p*, we considered three coupled master equations for the probability densities ${P}_{i}(\vec{r},\vec{e},t)$ to find a cell in state *i* at position $\vec{r}$, moving into the direction $\vec{e}$ at time *t*, where the state *i* denotes the pushing, pulling, or wrapped mode. By reduction to a diffusion equation for the cell density, we found the following expression for the diffusion coefficient of the swimmers,

1 $${\mathscr{D}}(p)=\frac{{v}_{0}^{2}}{\mathrm{3(2}+p)}.\frac{16{D}_{{\rm{\phi }}}(2{D}_{{\rm{\phi }}}+k)+4{D}_{{\rm{\phi }}}({D}_{{\rm{\phi }}}+3k)p+{k}^{2}{p}^{2}}{8{D}_{{\rm{\phi }}}[4{D}_{{\rm{\phi }}}^{2}+6{D}_{{\rm{\phi }}}k+{k}^{2}\mathrm{(2}+p)]}\mathrm{,}$$

with *v*_0_ the run speed during pushing and pulling and *D*_φ_ the rotational diffusion constant [for details of the derivation of Eq. (1) see Supplementary Notes Section I]. In the limit of *p* = 0, where the cells never switch into the wrapped mode and move in a classical push-pull-push pattern, Eq. (1) reduces to the familiar result for the diffusion coefficient of a swimmer with exponentially distributed reversal times,

2 $${\mathscr{D}}(p=0)=\frac{{v}_{0}^{2}}{\mathrm{6(}{D}_{{\rm{\phi }}}+k)}\,\mathrm{.}$$

**Figure 6:**
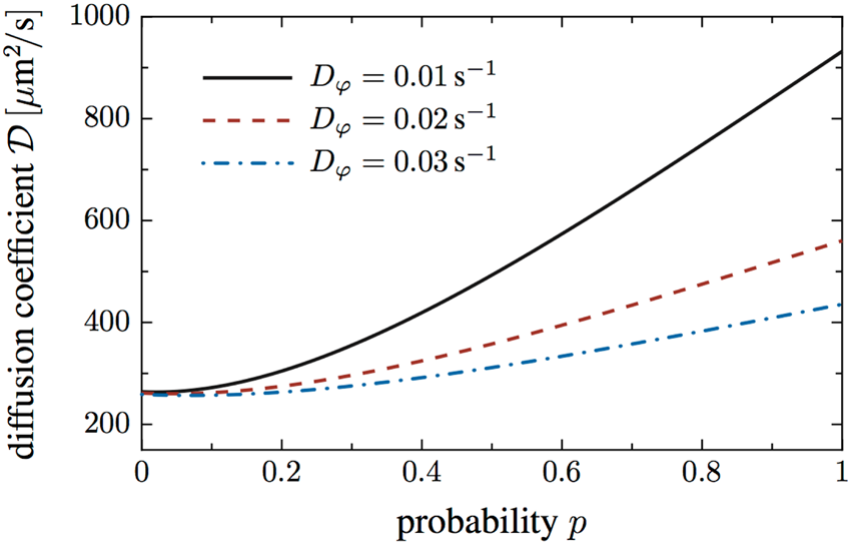
Diffusion coefficient as a function of the probability *p* that a transition from the pulling to the wrapped bundle configuration occurs. The diffusion coefficient of the swimmers is significantly enhanced in the presence of the wrapped mode, as displayed here for different values of the rotational diffusion constant.

Based on expression (1), we may now estimate the order of magnitude of the diffusion coefficient and can illustrate how the presence of the wrapped swimming mode affects the overall spreading of a population of swimmers. In Fig. 6, the diffusion coefficient of the swimmers is displayed as a function of the splitting probability *p* that expresses how likely a transition from the pulling to the wrapped bundle configuration occurs. Curves for three different values of the rotational diffusion constant are shown, the red dashed line being closest to the experimentally recorded value^33^. The remaining parameters were set to *k* ≈ 1*s* ^−1^ and *v*_0_ ≈ 40 *μ*m/s^33^. For lower rotational diffusion, in the range that is typical for *Pseudomonas putida*, the diffusion coefficient is significantly increased by the presence of the wrapped swimming state. Specifically, for a value of *D*_φ_ = 0.02 s^−1^, we find that the diffusion coefficient increases by a factor of two. In this estimate, we did not take the occasional stop events into account that were also observed in our data and are known to influence the navigation of other bacterial species, such as *Rhodobacter sphaeroides* ^28^.

Note that we cannot provide a quantitative test of these predictions with our present data. Our experimental setup was optimized to image the flagellar dynamics with high spatial and temporal resolution and thus contains only short parts of individual trajectories. To determine the diffusion coefficient together with the value of the splitting probability *p* requires mixed recordings in bright-field and fluorescent mode to obtain large numbers of long single cell trajectories together with sufficient information on the flagellar arrangement. This requires a novel experimental setting that has only recently been achieved for *E. coli* tracking experiments and will be the subject of our future research efforts^34^.

## Conclusion

We conclude that a bacterial swimmer that follows a push-and-pull pattern may strongly enhance its spreading by introducing a third low-speed swimming state into its motion cycle. Despite the low speed, this third state enables the cells to achieve longer excursions in a given direction, leading ultimately to a net increase in the diffusion coefficient. For the lophotrichiously flagellated soil bacterium *Pseudomonas putida*, this third state is associated with a torque-induced instability of the pulling flagella that initiates wrapping of the flagellar bundle around the cell body. In confined (pseudo one-dimensional) environments, where fluctuations of the bacterial body axis are strongly suppressed, our model also suggests increased excursions in a given direction due to the wrapped swimming mode, this time, however, leading to an effective bias and thus potentially to ballistic or super-diffusive motion. In general, for bacteria that navigate through a narrow porous environment, such as the soil bacterium *P. putida*, the wrapped mode may thus be an advantageous filament configuration. These observation are in agreement with the enhanced mean runlengths that we have previously observed for *P. putida* swimming in microchannels that were densely packed with cylindrical obstacles^12^. We therefore assume that the enhanced spreading, predicted by our model calculations in Fig. 6, also holds in *P. putida’s* natural soil habitat.

In summary, our results highlight that the wrapped filament configuration that has been recently reported for monotrichous *S. putrefaciens* under confinement^15^, is in fact a common mode of bacterial swimming. It robustly occurs across different bacterial species with varying flagellation patterns and serves distinct purposes not only for the escape from mechanical trapping but also for the efficient exploration of open fluid environments. An important open question is the role of this novel swimming mode for the chemotaxis strategy of bacteria that exhibit filament wrapping. In the case of soil bacteria, chemotaxis plays a vital role^35^, and also for *P. putida* several chemoattractants are known^36^. In the presence of a chemoattractant gradient we may envision, for example, that a net drift is induced by enhancing one of the swimming modes at the expense of the others. This aspect and related questions will be a focus of our future studies.

## Methods

### Enabling fluorescent staining of *P. putida* flagellar filaments

To enable coupling of fluorescent maleimide-ligated dyes to assembled flagellar filaments of *P. putida*, we introduced a surface-exposed cysteine residue to the building block of the filament, the protein flagellin (FliC, PP_4378). Serine 267 (Ser267) was selected for an appropriate serine to cysteine substitution based on flagellin sequence alignments and modeling of the *P. putida* flagellin structure similarly as described for *S. putrefaciens* ^15^. For the genetic substitution, flagellin-encoding *fliC* was first deleted and subsequently chromosomally replaced by the mutant variant by sequential homologous crossover essentially as previously described^15,37^. The corresponding genetic fusions or gene variants were constructed by PCR using appropriate primers and cloned into suicide vector pNPTS138-R6K by Gibson assembly^38^ in *E. coli* DH5*αλ*pir^39^. The vector was transferred to *P. putida* by conjugation with *E. coli* WM3064 (W. Metcalf, University of Indiana, Urbana, Champaign) as donor, using the standard protocol developed for *Shewanella* ^37^. All procedures were carried out on LB medium supplemented with 50 *μ*g/ml, 300 *μ*M 2,6-diaminoheptanedioic acid, and/or 10% (w/v) sucrose when appropriate. The swimming capacity of the resultant strain *P. putida* FliC_S267C_ was tested by determining the spreading of the mutant on 0.3% LB soft-agar plates directly compared to that of the wild type. Since no difference in motility on soft-agar plates occurred, we concluded that the Ser278Cys substitution does not negatively affect assembly and function of the flagellar filament.

### Bacterial cell culture

*P. putida* KT2440 FliC_S267C_ was grown in Tryptone broth (10 g/l tryptone (Applichem), 5 g/l NaCl) at 30 °C on a rotary shaker at 300 rpm. Cultures grown in Tryptone broth were incubated overnight to stationary phase and were directly used in this highly motile phase.

### Fluorescence staining

To couple thiol-reactive maleimide-ligated dyes to FliC_S267C_, we adapted the staining protocol from^40^. In short, 10 ml of cell suspension from overnight cultures was washed two times by centrifugation (4 min, 3500 g) and resuspended in motility buffer with glucose (11.2 g/1 K_2_HPO_4_, 4.8 g/1 KH_2_PO_4_, 3.93 g/l NaCl, 0.029 g/1 EDTA and 0.5 g/1 glucose; pH 7.0). During the last wash step the cell suspension was concentrated 20-fold to 0.5 ml in a 1.5 ml micro tube. 1 mg of Alexa 488 C5-maleimide was dissolved in 1ml DMSO of which 50 μl was added to the concentrated cell suspension. Cells were then incubated on a rocking shaker for 15 min at 40 rpm protected from light. To remove excess dye, the sample was washed again at least two times by centrifugation with replacement of the supernatant with fresh motility buffer. Motility was checked by eye under the microscope and was still present after up to 5 h. Addition of fresh Tryptone broth to a final concentration of 1:10 to the washed cell suspension in buffer resulted in an even higher motility and was used for some of the experiments.

### Image acquisition and analysis

All measurements were performed using an inverted wide field microscope (Olympus IX71) and recorded with a Hamamatsu Orca Flash 4.0 high speed camera. The objectives used were Olympus 40x, 60x, and 100x UPLFLN-PH. In order to achieve the required high speed fluorescence imaging of flagellar dynamics, we used a high powered blue LED as the excitation source (4.8W optical output power, 470 nm). Images were recorded on a PC running the Hokawo software (Hamamatsu) onto a SSD RAID. Up to a frame rate of 100 fps, a full field of view (2048 × 2048 px) was possible. For higher frame rates the field of view had to be reduced by half for each 100 fps speed increase.

Because of the high frame rates and sometime low densities of swimmers, recorded image stacks were processed with a custom ImageJ^41^ macro to extract only parts which contained swimmers. Our macro computed the grouped maximum projection for a batch of stacks. Each stack was divided into sub-sequences of 300 images each. Of those, the maximum intensity projection was computed, where moving cells will leave bright streaks in the projection of each sub-sequence. Those episodes were cropped in space and time to produce sub-stacks for further analysis. In this manner it was possible to reduce the large amount of data generated for visual inspection of all swimmers that could be recorded. Sub-stacks were then cataloged by swimming mode and observed transitions to obtain a complete picture of the motility pattern. Note that in most cases, also the cell body is slightly marked by the fluorescent dye, see the examples in Fig. 1. We attribute this to heterogeneities in the polysaccharide shell of the cells, due to which the dye can access proteins in the outer cell membrane to a degree that varies from cell to cell.

For several episodes of swimming in pushing, pulling, and wrapped mode mean speeds of propagation were computed by measuring the path length from the maximum intensity projection and noting beginning and end frames. The bundle rotation frequency of wrapped swimmers was measured using ImageJ’s orthogonal views^41^. This essentially computes kymographs along two orthogonal lines, which we drew through the cell body. By measuring the periodic brightness fluctuations in these projections, we calculated the rotation frequency for several wrapped swimmers. Helix pitch and wave length of the flagella were measured by hand from still frames in which the bundle was clearly visible and parallel to the focal plane.

### Data availability

The datasets generated and analysed during the current study are available from the corresponding author on reasonable request.

## Electronic supplementary material

Supplementary Information

Swimming as a pusher

Swimming as a puller

Transition from pushing to pulling

Swimming with the flagella wrapped around the cell body

Handedness of the flagella in wrapped mode

Swimming as a pusher with frequent stops

Swimming as a pusher with loose bundle interrupted by a stop

Transition from pulling to pushing

Transition from a pulling to a wrapped bundle

Transition from a pulling to a wrapped bundle of an immobilized cell

Transitions from a pulling to a wrapped bundle and from a wrapped to a pushing bundle

Transition from a wrapped to a pushing bundle of an immobilized cell

Transitions without change in the polymorphic state of the flagella

## References

[1] Bray, D. *Cell Movements*: *From Molecules to Motility*, 2nd edn, (Garland Science, New York, 2000).

[2] Berg, H. C. *E. coli in Motion*, 1st edn (Springer, New York, 2004).

[3] Turner L, Ryu WS, Berg HC (2000). Real-Time Imaging of Fluorescent Flagellar Filaments. J Bacteriol.

[4] Taylor BL, Koshland DE (1974). Reversal of Flagellar Rotation in Monotrichous and Peritrichous Bacteria: Generation of Changes in Direction. J Bacteriol.

[5] Johansen JE, Pinhassi J, Blackburn N, Zweifel UL, Hagström A (2002). Variability in motility characteristics among marine bacteria. Aquat Microb Ecol.

[6] Xie L, Altindal T, Chattopadhyay S, Wu X-L (2011). Bacterial flagellum as a propeller and as a rudder for efficient chemotaxis. PNAS.

[7] Son K, Guasto JS, Stocker R (2013). Bacteria can exploit a flagellar buckling instability to change direction. Nat Phys.

[8] Theves M, Taktikos J, Zaburdaev V, Stark H, Beta C (2013). A bacterial swimmer with two alternating speeds of propagation. Biophysical journal.

[9] Harwood CS, Fosnaugh K, Dispensa M (1989). Flagellation of pseudomonas putida and analysis of its motile behavior. J Bacteriol.

[10] Macnab RM (1977). Bacterial flagella rotating in bundles: a study in helical geometry. Proceedings of the National Academy of Sciences.

[11] Kim M, Bird JC, Van Parys AJ, Breuer KS, Powers TR (2003). A macroscopic scale model of bacterial flagellar bundling. Proceedings of the National Academy of Sciences.

[12] Raatz M (2015). Swimming patterns of a polarly flagellated bacterium in environments of increasing complexity. Eur. Phys. J. Special Topics.

[13] Lauga E, Powers TR (2009). The hydrodynamics of swimming microorganisms. Rep. Prog. Phys..

[14] Lauga E, DiLuzio WR, Whitesides GM, Stone HA (2006). Swimming in circles: Motion of bacteria near solid boundaries. Biophys. J..

[15] Kühn MJ, Schmidt FK, Eckhardt B, Thormann KM (2017). Bacteria exploit a polymorphic instability of the flagellar filament to escape from traps. PNAS.

[16] Asakura S (1970). Polymerization of flagellin and polymorphism of flagella. Adv. Biophys..

[17] Calladine CR (1978). Change of waveform in bacterial flagella: the role of mechanics at the molecular level. Journal of Molecular Biology.

[18] Darnton NC, Berg HC (2007). Force-Extension Measurements on Bacterial Flagella: Triggering Polymorphic Transformations. Biophys J.

[19] Vogel R, Stark H (2010). Force-extension curves of bacterial flagella. The European Physical Journal E.

[20] Berg HC (1975). Chemotaxis in bacteria. Annual review of biophysics and bioengineering.

[21] Mears PJ, Koirala S, Rao CV, Golding I, Chemla YR (2014). Escherichia coli swimming is robust against variations in flagellar number. eLife.

[22] Bray D, Levin MD, Morton-Firth CJ (1998). Receptor clustering as a cellular mechanism to control sensitivity. Nature.

[23] Duke TAJ, Bray D (1999). Heightened sensitivity of a lattice of membrane receptors. Proceedings of the National Academy of Sciences.

[24] Vladimirov N, Løvdok L, Lebiedz D, Sourjik V (2008). Dependence of Bacterial Chemotaxis on Gradient Shape and Adaptation Rate. PLOS Computational Biology.

[25] Bray D (2013). The propagation of allosteric states in large multiprotein complexes. Journal of Molecular Biology.

[26] Murat D (2015). Opposite and coordinated rotation of amphitrichous flagella governs oriented swimming and reversals in a magnetotactic spirillum. Journal of Bacteriology.

[27] Ping L, Birkenbeil J, Monajembashi S (2013). Swimming behavior of the monotrichous bacterium Pseudomonas fluorescens SBW25. FEMS Microbiol Ecol.

[28] Armitage JP, Macnab RM (1987). Unidirectional, intermittent rotation of the flagellum of Rhodobacter sphaeroides. J Bacteriol.

[29] Vogel R, Stark H (2013). Rotation-induced polymorphic transitions in bacterial flagella. Phys. Rev. Lett..

[30] Taktikos J, Stark H, Zaburdaev V (2013). How the Motility Pattern of Bacteria Affects Their Dispersal and Chemotaxis. PLoS ONE.

[31] Großmann R, Peruani F, Bär M (2016). Diffusion properties of active particles with directional reversal. New J. Phys..

[32] Großmann R, Peruani F, Bär M (2015). A geometric approach to self-propelled motion in isotropic & anisotropic environments. Eur. Phys. J.: Spec. Top..

[33] Theves M, Taktikos J, Zaburdaev V, Stark H, Beta C (2015). Random walk patterns of a soil bacterium in open and confined environments. EPL (Europhysics Letters).

[34] Turner L, Ping L, Neubauer M, Berg HC (2016). Visualizing flagella while tracking bacteria. Biophysical Journal.

[35] Webb BA (2017). Sinorhizobium meliloti chemotaxis to quaternary ammonium compounds is mediated by the chemoreceptor McpX. Molecular Microbiology.

[36] Harwood CS, Parales RE, Dispensa M (1990). Chemotaxis of Pseudomonas putida toward chlorinated benzoates. Applied and environmental microbiology.

[37] Lassak J, Henche A-L, Binnenkade L, Thormann KM (2010). ArcS, the Cognate Sensor Kinase in an Atypical Arc System of Shewanella oneidensis MR-1. Appl Environ Microbiol.

[38] Gibson DG (2009). Enzymatic assembly of DNA molecules up to several hundred kilobases. Nat Meth.

[39] Miller VL, Mekalanos JJ (1988). A novel suicide vector and its use in construction of insertion mutations: osmoregulation of outer membrane proteins and virulence determinants in Vibrio cholerae requires toxR. J Bacteriol.

[40] Turner L, Zhang R, Darnton NC, Berg HC (2010). Visualization of flagella during bacterial swarming. Journal of Bacteriology.

[41] Schneider CA, Rasband WS, Eliceiri KW (2012). NIH image to ImageJ: 25 years of image analysis. Nature Methods.

