# Author Correction: A polar bundle of flagella can drive bacterial swimming by pushing, pulling, or coiling around the cell body

**DOI:** 10.1038/s41598-022-16722-1

**Published:** 2022-08-12

**Authors:** Marius Hintsche, Veronika Waljor, Robert Großmann, Marco J. Kühn, Kai M. Thormann, Fernando Peruani, Carsten Beta

**Affiliations:** 1 University of Potsdam, Institute of Physics and Astronomy, 14476 Potsdam, Germany; 2 Université Côte d’Azur, Laboratoire J. A. Dieudonné, UMR 7351 CNRS, 06108 Nice Cedex 02, France; 3 Institut Für Mikrobiologie Und Molekularbiologie, Justus-Liebig-Universität Giessen, 35392 Giessen, Germany

Correction to: Scientific Reports, 10.1038/s41598-017-16428-9, published on 1 December 2017

This Article contains an error in the legend of Figure 2. As a result,

“*P. putida* can swim with the flagellar bundle wrapped around the cell body. (**A**) Fluorescence image and (**B**) cartoon of the wrapped mode. The cell swims in the direction indicated by the arrow – the posterior (flagellated) pole of the cell points in swimming direction, see also the Supplementary Videos S4 and S5. (**C**) Percentages of swimming states observed in our data set (out of 794 runs in total). Scale bar is 5 *μ*m.”

should read:

“*P. putida* can swim with the flagellar bundle wrapped around the cell body. (**A**) Fluorescence image and (**B**) cartoon of the wrapped mode. The cell swims in the direction indicated by the arrow – the posterior (flagellated) pole of the cell points in swimming direction, see also the Supplementary Videos S4 and S5. (**C**) Percentages of swimming states observed in our data set (out of 794 runs in total). Scale bar is 2 *μ*m.”

