# Supplementary material for: A polar bundle of flagella can drive bacterial swimming by pushing, pulling, or coiling around the cell body
Source: Sci Rep. 2017 Dec 1;7:16771. doi: 10.1038/s41598-017-16428-9 (PMC5711944; doi:10.1038/s41598-017-16428-9)
Supplement: Supplementary file 1 — Supplementary Information [file 41598_2017_16428_MOESM1_ESM.pdf]

SUPPLEMENTARY INFORMATION

A polar bundle of flagella can drive bacterial swimming by pushing, pulling, or coiling around the cell body

by Marius Hintsche, Veronika Waljor, Robert Großmann, Marco J. Kühn,
Kai M. Thormann, Fernando Peruani, and Carsten Beta

I. DIFFUSION COEFFICIENT OF *PSEUDOMONAS PUTIDA*

INTRODUCTION

The derivation of the diffusion coefficient of an active particle with *push-pull-wrapped* motility states is briefly sketched. Basically, the idea of the derivation is to reduce the Master equation, that accounts for the full dynamics, to the diffusion equation

$$\partial_t \rho(\mathbf{r},t) = \mathcal{D}\Delta\rho(\mathbf{r},t), \tag{S1}$$

which is valid at large scales in the long-time limit. The diffusion coefficient $\mathcal{D}$ can be read off from (S1).

THE MODEL

The starting point of the derivation is the following Master equation for the probability densities $P_i(\mathbf{r},\mathbf{e},t)$ to find a particle in the motion state i at position $\mathbf{r}$, moving in direction $\mathbf{e}$ at time t:

$$\partial_t P_1(\mathbf{r},\mathbf{e},t) = -v_0\,\mathbf{e}\cdot\nabla P_1(\mathbf{r},\mathbf{e},t) + \mathcal{L}_\varphi[P_1(\mathbf{r},\mathbf{e},t)] - kP_1(\mathbf{r},\mathbf{e},t) + kP_3(\mathbf{r},-\mathbf{e},t) + k(1-p)P_2(\mathbf{r},-\mathbf{e},t), \tag{S2a}$$

$$\partial_t P_2(\mathbf{r},\mathbf{e},t) = -v_0\,\mathbf{e}\cdot\nabla P_2(\mathbf{r},\mathbf{e},t) + \mathcal{L}_\varphi[P_2(\mathbf{r},\mathbf{e},t)] - kP_2(\mathbf{r},\mathbf{e},t) + kP_1(\mathbf{r},-\mathbf{e},t), \tag{S2b}$$

$$\partial_t P_3(\mathbf{r},\mathbf{e},t) = -\frac{v_0}{2}\mathbf{e}\cdot\nabla P_3(\mathbf{r},\mathbf{e},t) + \mathcal{L}_\varphi[P_3(\mathbf{r},\mathbf{e},t)] - kP_3(\mathbf{r},\mathbf{e},t) + kpP_2(\mathbf{r},\mathbf{e},t). \tag{S2c}$$

We assign the following indices to the respective motility patterns: (1) *push*, (2) *pull*, (3) *wrapped*. The first terms on the right hand side describe the convective transport of the particles along $\mathbf{e}$ at the speed of the corresponding motility state. Notice that the speed in the *wrapped* state (3) is assumed to be a factor of two smaller compared to the *push* (1) and *pull* (2) state, which are considered to be equal.

The Fokker-Planck operators $\mathcal{L}_\varphi[P_i]$ in Eqs. (S2) account for random reorientations of the body axis due to spatial heterogeneities or fluctuations of the driving motor inducing rotational diffusion. Fluctuations may change the direction of motion, however, the length of the unit vector $\mathbf{e}$ is preserved. Accordingly, the Langevin dynamics of the director $\mathbf{e}$ driven by Gaussian white noise $\boldsymbol{\xi}(t)$ reads [1, 2]

$$\dot{e}_\mu(t) = \sqrt{2D_\varphi}\left[\delta_{\mu\nu} - e_\mu(t)\,e_\nu(t)\right]\xi_\nu(t), \tag{S3}$$

which is to be interpreted in the sense of Stratonovich [2]. Throughout, Einstein notation is used, *i.e.*, repeated indices is summed over. The Fokker-Planck operator corresponding to the Langevin equation (S3) yields

$$\mathcal{L}_\varphi[P_i(\mathbf{r},\mathbf{e},t)] = D_\varphi\left\{(d-1)\frac{\partial}{\partial e_\mu}\left[e_\mu P_i(\mathbf{r},\mathbf{e},t)\right] + \frac{\partial^2}{\partial e_\mu \partial e_\nu}\left[(\delta_{\mu\nu} - e_\mu e_\nu)P_i(\mathbf{r},\mathbf{e},t)\right]\right\}, \tag{S4}$$

where D_φ denotes the rotational diffusion coefficient, which parametrizes the intensity of fluctuations experienced on the moving direction $\mathbf{e}$ and d is the spatial dimension.

The remaining terms in Eqs. (S2) describe the transitions between the motion states. The mean time τ that is spent in each of these states is parametrized by $\tau = k^{-1}$ which is assumed to be equal for all states. The transitions *push* (1) $\leftrightarrow$ *pull* (2) as well as *wrapped* (3) $\rightarrow$ *push* (1) are accompanied by a reversal event ($\mathbf{e} \rightarrow -\mathbf{e}$), whereas no reorientation is assumed to occur for the transition *pull* (2) $\rightarrow$ *wrapped* (3). Additionally, we introduce the splitting probability p leaving the *pull* (2) state: the transition *pull* (2) $\rightarrow$ *wrapped* (3) occurs with a probability p whereas the transition *pull* (2) $\rightarrow$ *push* (1) is observed with probability $1-p$. Hence, the model effectively reduces to a two-state dynamics with the transitions

push (1) $\leftrightarrow$ *pull* (2)

for $p = 0$, whereas it becomes equivalent to a *clock model* [3] for $p = 1$ with the linear sequence of states

$$\textit{push}\ (1) \rightarrow \textit{pull}\ (2) \rightarrow \textit{wrapped}\ (3) \rightarrow \textit{push}\ (1)\ ...$$

REDUCTION TO THE DIFFUSION EQUATION

In order to obtain the dynamics of the density $\rho(\mathbf{r}, t)$, we perform a moment expansion of the Master equation. At first, the density components

$$\rho_i(\mathbf{r}, t) = \int d^d e\, P_i(\mathbf{r}, \mathbf{e}, t) \tag{S5}$$

are considered. Their dynamics is derived by integrating Eqs. (S2) over the values of the director $\mathbf{e}$ which yields

$$\partial_t \rho_1 = -v_0 \nabla \cdot \mathbf{w}_1 - k\rho_1 + k\rho_3 + k(1-p)\rho_2, \tag{S6a}$$
$$\partial_t \rho_2 = -v_0 \nabla \cdot \mathbf{w}_2 - k\rho_2 + k\rho_1, \tag{S6b}$$
$$\partial_t \rho_3 = -\frac{v_0}{2} \nabla \cdot \mathbf{w}_3 - k\rho_3 + kp\rho_2, \tag{S6c}$$

where $\mathbf{w}_i(\mathbf{r}, t)$ represents the mean local orientation (of motility state i), defined as the first moment of the probability density $P_i(\mathbf{r}, \mathbf{e}, t)$:

$$\mathbf{w}_i(\mathbf{r}, t) = \int d^d e\, \mathbf{e}\, P_i(\mathbf{r}, \mathbf{e}, t). \tag{S7}$$

Notice that in Eq. (S6) the arguments $\{\mathbf{r}, t\}$ were omitted for simplicity. The dynamics of the total density

$$\rho = \rho_1 + \rho_2 + \rho_3 \tag{S8}$$

follows in a straightforward way from the sum of Eqs. (S6):

$$\partial_t \rho = -v_0 \left[\nabla \cdot \mathbf{w}_1 + \nabla \cdot \mathbf{w}_2 + \frac{1}{2} \nabla \cdot \mathbf{w}_3 \right] = -v_0 \left(1, 1, \frac{1}{2} \right) \cdot \begin{pmatrix} \nabla \cdot \mathbf{w}_1 \\ \nabla \cdot \mathbf{w}_2 \\ \nabla \cdot \mathbf{w}_3 \end{pmatrix}. \tag{S9}$$

Upon summation, all transition terms (those proportional to k) cancel. Hence, the total density $\rho(\mathbf{r}, t)$ is a hydrodynamic mode whose dynamics is much slower compared to all the other moments of the probability densities $P_i(\mathbf{r}, \mathbf{e}, t)$ (see below) and thus allowing to reduce the dynamics solely to the density in the long-time limit. Notice, however, that the density dynamics is not closed but coupled to $\mathbf{w}_i(\mathbf{r}, t)$. Moreover, the dynamics of the fields $\mathbf{w}_i(\mathbf{r}, t)$ is coupled to higher order moments giving rise to an infinite hierarchy. Despite of this, we can make use of the fact that the dynamics of higher modes is fast, *i.e.*, their dynamics is slaved [4] to the density in the long time limit thus allowing for their adiabatic elimination. Furthermore, we keep in mind that we want to reduce the dynamics to the diffusion equation. Thus, it is sufficient to calculate the respective moments to first order in density gradients in the long-time limit.

The dynamics of the first moment $\mathbf{w}_i(\mathbf{r}, t)$ is obtained by multiplication of Eqs. (S2) with $\mathbf{e}$ and subsequent integration:

$$\partial_t \mathbf{w}_1 = -v_0 \nabla \cdot \hat{\mathcal{T}}_1 - \big[D_\varphi (d-1) + k \big] \mathbf{w}_1 - k\mathbf{w}_3 - k(1-p)\mathbf{w}_2\,, \tag{S10a}$$
$$\partial_t \mathbf{w}_2 = -v_0 \nabla \cdot \hat{\mathcal{T}}_2 - \big[D_\varphi (d-1) + k \big] \mathbf{w}_2 - k\mathbf{w}_1\,, \tag{S10b}$$
$$\partial_t \mathbf{w}_3 = -\frac{v_0}{2} \nabla \cdot \hat{\mathcal{T}}_3 - \big[D_\varphi (d-1) + k \big] \mathbf{w}_3 + kp\mathbf{w}_2\,. \tag{S10c}$$

To obtain a closed set of equations, we iterate the moment expansion once more, deriving the dynamics of the symmetric tensors

$$\left\{ \hat{\mathcal{T}}_i(\mathbf{r}, t) \right\}_{\mu\nu} = \int d^d e\, e_\mu e_\nu P_i(\mathbf{r}, \mathbf{e}, t) \tag{S11}$$

given by

$$\partial_t \hat{\mathcal{T}}_1 \simeq 2D_\varphi \rho_1 \mathbb{1} - \big[2dD_\varphi + k \big] \hat{\mathcal{T}}_1 + k\hat{\mathcal{T}}_3 + k(1-p)\hat{\mathcal{T}}_2\,, \tag{S12a}$$
$$\partial_t \hat{\mathcal{T}}_2 \simeq 2D_\varphi \rho_2 \mathbb{1} - \big[2dD_\varphi + k \big] \hat{\mathcal{T}}_2 + k\hat{\mathcal{T}}_1\,, \tag{S12b}$$
$$\partial_t \hat{\mathcal{T}}_3 \simeq 2D_\varphi \rho_3 \mathbb{1} - \big[2dD_\varphi + k \big] \hat{\mathcal{T}}_3 + kp\hat{\mathcal{T}}_2\,. \tag{S12c}$$

The coupling to higher order modes was neglected in the last step as the resulting terms are irrelevant in the diffusive limit.
Now, we reduce Eqs. (S10) and Eqs. (S12) onto the density $\rho(\mathbf{r},t)$, which is the only slow degree of freedom, by adiabatic elimination. This implies that we solve these equations in the stationary state, and thus assume that $\partial_t \mathbf{w}_i(\mathbf{r},t) \simeq 0$ and $\partial_t \hat{\mathcal{T}}_i(\mathbf{r},t) \simeq 0$. To do so, we first express the density components $\rho_i(\mathbf{r},t)$ in terms of the total density $\rho(\mathbf{r},t)$. In the long-time limit, the densities $\rho_i(\mathbf{r},t)$ are proportional to the density $\rho(\mathbf{r},t)$ times the probability to find a particle in one of the motion states:

$$\begin{pmatrix}\rho_1(\mathbf{r},t)\\ \rho_2(\mathbf{r},t)\\ \rho_3(\mathbf{r},t)\end{pmatrix} \simeq \rho(\mathbf{r},t)\cdot \begin{pmatrix}1/(2+p)\\ 1/(2+p)\\ p/(2+p)\end{pmatrix}. \tag{S13}$$

For $p=0$, the particle is found with the probability $1/2$ in the *push* (1) and *pull* (2) states, respectively, whereas the probability to be in the *push* (1), *pull* (2) or *wrapped* (3) mode equals $1/3$ in the limit $p=1$. By inserting the solution (S13) into Eqs. (S12), we can conclude that the off-diagonal elements of the tensors $\hat{\mathcal{T}}_i(\mathbf{r},t)$ vanish and, further, all diagonal elements are equal. In short, we find $\left\{\hat{\mathcal{T}}_i(\mathbf{r},t)\right\}_{\mu\nu} = t_i(\mathbf{r},t)\,\delta_{\mu\nu}$ where

$$\begin{pmatrix}t_1(\mathbf{r},t)\\ t_2(\mathbf{r},t)\\ t_3(\mathbf{r},t)\end{pmatrix} \simeq \frac{\rho(\mathbf{r},t)}{d}\cdot \begin{pmatrix}1/(2+p)\\ 1/(2+p)\\ p/(2+p)\end{pmatrix}. \tag{S14}$$

Eventually, we eliminate $\mathbf{w}_i(\mathbf{r},t)$ by solving Eqs. (S10) to obtain the following closure for $\mathbf{w}_i(\mathbf{r},t)$ to first order in spatial gradients:

$$\begin{pmatrix}\mathbf{w}_1(\mathbf{r},t)\\ \mathbf{w}_2(\mathbf{r},t)\\ \mathbf{w}_3(\mathbf{r},t)\end{pmatrix} \simeq -\frac{v_0}{d}\begin{pmatrix}D_\varphi(d-1)+k & k(1-p) & k\\ k & D_\varphi(d-1)+k & 0\\ 0 & -kp & D_\varphi(d-1)+k\end{pmatrix}^{-1}\cdot \begin{pmatrix}\big(1/(2+p)\big)\nabla\rho(\mathbf{r},t)\\ \big(1/(2+p)\big)\nabla\rho(\mathbf{r},t)\\ \big(p/(4+2p)\big)\nabla\rho(\mathbf{r},t)\end{pmatrix}. \tag{S15}$$

Combining Eqs. (S9) with this constitutive relation yields the diffusion equation

$$\partial_t\rho(\mathbf{r},t) \simeq \left[\frac{v_0^2}{d}\cdot\left(1,1,\frac{1}{2}\right)\cdot\begin{pmatrix}D_\varphi(d-1)+k & k(1-p) & k\\ k & D_\varphi(d-1)+k & 0\\ 0 & -kp & D_\varphi(d-1)+k\end{pmatrix}^{-1}\cdot\begin{pmatrix}1/(2+p)\\ 1/(2+p)\\ p/(4+2p)\end{pmatrix}\right]\Delta\rho(\mathbf{r},t). \tag{S16}$$

We emphasize that the expression for the diffusion coefficient derived above is asymptotically exact even though the reduction of the full dynamics [Eqs. (S2)] onto the diffusion equation involved approximations as short-time processes were neglected.

DIFFUSION COEFFICIENT

From the diffusion equation (S16) one may easily read off the diffusion coefficient

$$\mathcal{D} = \frac{v_0^2}{d}\cdot\left(1,1,\frac{1}{2}\right)\cdot\begin{pmatrix}D_\varphi(d-1)+k & k(1-p) & k\\ k & D_\varphi(d-1)+k & 0\\ 0 & -kp & D_\varphi(d-1)+k\end{pmatrix}^{-1}\cdot\begin{pmatrix}1/(2+p)\\ 1/(2+p)\\ p/(4+2p)\end{pmatrix} \tag{S17}$$

which yields after some straightforward matrix algebra in two dimensions ($d=2$)

$$\mathcal{D} = \frac{v_0^2}{2(2+p)}\cdot\frac{8D_\varphi(D_\varphi+k)+D_\varphi(D_\varphi+6k)\,p+k^2p^2}{4D_\varphi\big[D_\varphi^2+3D_\varphi k+k^2(2+p)\big]}, \tag{S18}$$

or, alternatively, in three dimensions ($d=3$)

$$\mathcal{D} = \frac{v_0^2}{3(2+p)}\cdot\frac{16D_\varphi(2D_\varphi+k)+4D_\varphi(D_\varphi+3k)\,p+k^2p^2}{8D_\varphi\big[4D_\varphi^2+6D_\varphi k+k^2(2+p)\big]}. \tag{S19}$$

In the limit $p=0$, where the *wrapped* (3) state is never adopted by the particle, these expressions reduce to the familiar result for the diffusion coefficient of an active particle with exponentially distributed reversal times:

$$\lim_{p\to 0}\mathcal{D} = \frac{v_0^2}{d\big[D_\varphi(d-1)+2k\big]}. \tag{S20}$$

We use the expression for the diffusion coefficient to estimate its order of magnitude and to illustrate the consequences of the existence of the *wrapped* (3) state. The diffusion coefficient as a function of the probability p that a transition *pull* (2) $\rightarrow$ *wrapped* (3) occurs is represented in Fig. 6 of the main text for several values of the rotational diffusion coefficient D_φ. The remaining parameters were estimated to be [5]

$$k \approx 1\,\mathrm{s}^{-1}, \quad v_0 \approx 40\,\frac{\mu\mathrm{m}}{s}. \tag{S21}$$

For low rotational diffusion, which is typical for *Pseudomonas putida*, the diffusion coefficient is significantly increased by the presence of a third swimming state, *i.e.*, the *wrapped* (3) state.

II. COMPARISON OF RUN SPEEDS IN DIFFERENT SWIMMING MODES

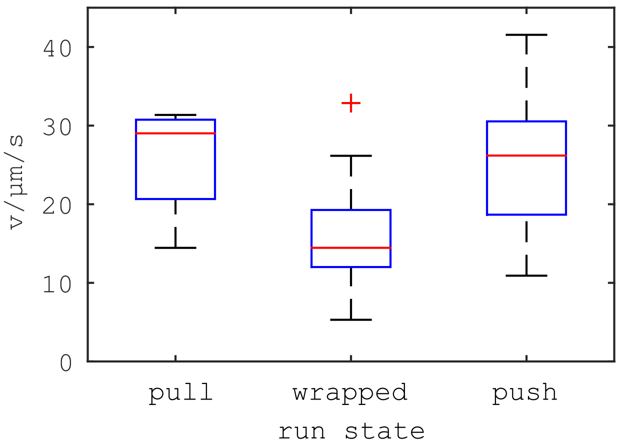

FIG. S1. Comparison of run speeds in pulling, wrapped, and pushing mode. From 31 trajectories with sufficient resolution, 61 run speeds were measured to calculated the mean speeds in the three different swimming modes.

III. TRANSITION SCENARIOS IMAGED ON IMMOBILIZED CELLS

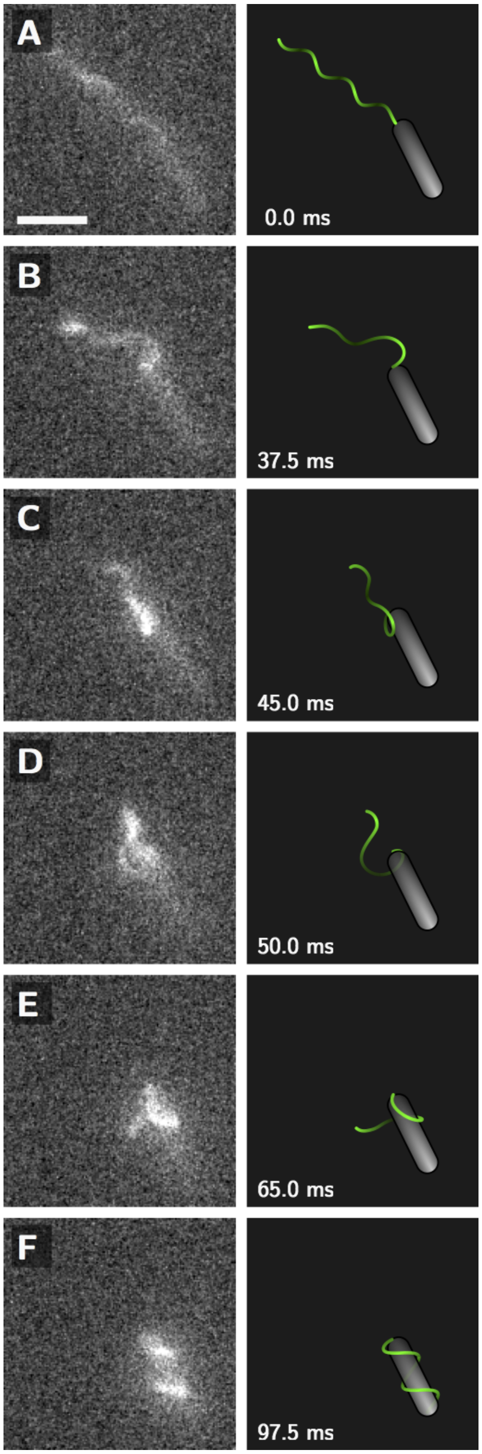

FIG. S2. Transition from a pulling to a wrapped boundle visualized on an immobilized cell. (Left) Snapshots of the transition from a fluorescence microscopy recording. The cell body is stuck to the cover slip, but the flagellum can rotate freely. Scale bar is 3 μm. (Right) Cartoon of the transition. See also Supplementary Video S10 online.

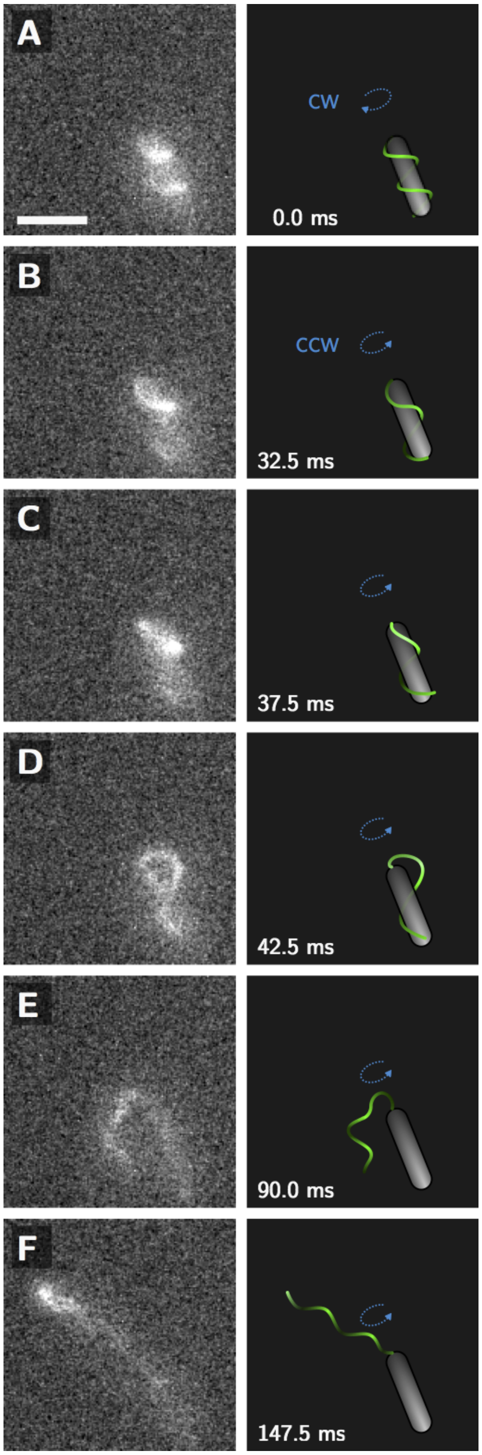

FIG. S3. Transition from a wrapped to a pushing boundle visualized on an immobilized cell. (Left) Snapshots of the transition from a fluorescence microscopy recording. The cell body is stuck to the cover slip, but the flagellum can rotate freely. Blue arrows mark the sense of motor rotation, which changes immediately before panel (B). Scale bar is 3 μm. (Right) Cartoon of the transition. See also Supplementary Video S12 online.

IV. SUPPLEMENTARY VIDEOS

Video S1: Swimming as a pusher. Example of a cell that swims in pushing mode. Close to the cover slip, the trajectory is curved to the right hand side.

Video S2: Swimming as a puller. Example of a cell that swims in pulling mode. Close to the cover slip, the trajectory is curved to the left hand side.

Video S3: Transition from pushing to pulling. Example of a push-pull transition. Before the motors reverse, their rotation shortly stops and the bundle disassembles, clearly showing that it is composed of several flagella.

Video S4: Swimming with the flagella wrapped around the cell body. Example of a cell that swims in wrapped mode.

Video S5: Handedness of the flagella in wrapped mode. Example of a cell that swims in wrapped mode. Upon entering the plane of focus from above, we can clearly determine the handedness of the flagella. Note that the apparent handedness depends on the position of the focal plane relative to the helix. A left-handed helix appears left-handed when the cell is below the focal plane and right-handed when the cell is above the focal plane. Here, we are imaging close to the cover slip, i.e. a cell that is approaching from the bulk fluid enters the focal plane from above. From the right-handed appearance of the helix in this case, we can conclude that the wrapped bundle forms a left-handed helix.

Video S6: Swimming as a pusher with frequent stops. Example of a cell that swims in pushing mode, interrupted by several stops. During the stop events, the flagellar bundle partly disassembles.

Video S7: Swimming as a pusher with loose bundle interrupted by a stop. Example of a cell that swims with a loose bundle in pushing mode, interrupted by a stop. During the stop events, the flagellar bundle disassembles.

Video S8: Transition from pulling to pushing. Example of a pull-push transition.

Video S9: Transition from a pulling to a wrapped bundle. Example of a cell that swims as a puller and switches to the wrapped mode, where its flagella are tightly coiled around the cell body. Snapshots of this video are displayed in Fig. 3 of the main text.

Video S10: Transition from a pulling to a wrapped bundle of an immobilized cell. Example of a transition from pulling to wrapped mode for a cell that was accidentially immobilized at the cover slip. Snapshots of this video are displayed in Supplementary Fig. S2 online.

Video S11: Transitions from a pulling to a wrapped bundle and from a wrapped to a pushing bundle. Example of a cell that undergoes a transition from pulling to wrapped mode followed by a transition from wrapped to pushing mode. Snapshots of the second transition from wrapped to pushing mode are displayed in Fig. 4 of the main text.

Video S12: Transition from a wrapped to a pushing bundle of an immobilized cell. Example of a transition from wrapped to pushing mode for a cell that was accidentially immobilized at the cover slip. Snapshots of this video are displayed in Supplementary Fig. S3 online.

Video S13: Transitions without change in the polymorphic state of the flagella. Example of an immobilized cell that undergoes a sequence of transitions wrapped-push-pull-wrapped without changing the polymorphic state of the flagella. The bundle remains in the coiled state throughout the recording. See the Supplementary Videos S10 and S12 for comparison, where the transitions are associated with a change between coiled and normal polymorphic forms.

[1] R. Großmann, F. Peruani, and M. Bär, Eur. Phys. J.: Spec. Top. **224**, 1377 (2015).
[2] C. Gardiner, *Stochastic Methods: A Handbook for the Natural and Social Sciences*, Springer Series in Synergetics (Springer, 2009).
[3] R. Großmann, F. Peruani, and M. Bär, New J. Phys. **18**, 043009 (2016).
[4] H. Haken, *Synergetics: Introduction and Advanced Topics* (Springer, 2004).
[5] M. Theves, J. Taktikos, V. Zaburdaev, H. Stark, and C. Beta, Europhys. Lett. **109**, 28007 (2015).
[6] E. Bertin, M. Droz, and G. Grégoire, Phys. Rev. E **74**, 022101 (2006).
[7] R. Großmann, L. Schimansky-Geier, and P. Romanczuk, New J. Phys. **15**, 085014 (2013).
